# Concordance in hippocampal and fecal *Nr3c1* methylation is moderated by maternal behavior in the mouse

**DOI:** 10.1002/ece3.416

**Published:** 2012-11-08

**Authors:** Shayna A Liberman, Rahia Mashoodh, Robert C Thompson, Dana C Dolinoy, Frances A Champagne

**Affiliations:** 1 Department of Environmental Health Sciences, University of Michigan School of Public HealthAnn Arbor, Michigan, 48109; 2 Department of Psychology, Columbia UniversityNew York, New York, 10027; 3 Department of Psychiatry, University of MichiganAnn Arbor, Michigan, 48109

**Keywords:** DNA methylation, fecal samples, glucocorticoid receptor, maternal behavior, mouse

## Abstract

Recent advances in genomic technologies now enable a reunion of molecular and evolutionary biology. Researchers investigating naturally living animal populations are thus increasingly able to capitalize upon genomic technologies to connect molecular findings with multiple levels of biological organization. Using this vertical approach in the laboratory, epigenetic gene regulation has emerged as an important mechanism integrating genotype and phenotype. To connect phenotype to population fitness, however, this same vertical approach must now be applied to naturally living populations. A major obstacle to studying epigenetics in noninvasive samples is tissue specificity of epigenetic marks. Here, using the mouse as a proof-of-principle model, we present the first known attempt to validate an epigenetic assay for use in noninvasive samples. Specifically, we compare DNA methylation of the NGFI-A (nerve growth factor-inducible protein A) binding site in the promoter of the glucocorticoid receptor (*Nr3c1*) gene between central (hippocampal) and peripheral noninvasive (fecal) tissues in juvenile Balb/c mice that had received varying levels of postnatal maternal care. Our results indicate that while hippocampal DNA methylation profiles correspond to maternal behavior, fecal DNA methylation levels do not. Moreover, concordance in methylation levels between these tissues within individuals only emerges after accounting for the effects of postnatal maternal care. Thus, although these findings may be specific to the *Nr3c1* gene, we urge caution when interpreting DNA methylation patterns from noninvasive tissues, and offer suggestions for further research in this field.

## Introduction

With the advent of genomic resources, reuniting molecular and evolutionary biology has become the subject of intensive research investigation (Dean and Thornton 2007). Such efforts have led to a deeper understanding of the relationship between genotype, phenotype, and fitness (Bradley and Lawler 2011). This vertical and integrative approach is especially useful for assessing natural populations, in which the use of whole-organism performance (made up of physiological, morphological, and behavioral phenotypes) can be used to determine fitness. Understanding the evolutionary trajectory of a species requires an understanding of both its genetic makeup and its ecology (behavior and environment). Although this model was implicated in the modern synthesis, only recently has the technology been available to concurrently examine multiple levels of biological organization (Dalziel et al. 2009). Indeed, a rapidly expanding body of theoretical and empirical literature spanning multiple disciplines has recently emerged (Ellegren and Sheldon 2008; Piertney and Webster 2010; Bradley and Lawler 2011; Martin et al. 2011).

One incipient theme in this “revolution” is the role of epigenetics in mediating the relationship between genotype and phenotype. Epigenetics is the study of mitotically (and potentially meiotically) heritable molecular modifications to DNA and chromatin that do not involve alteration to the underlying DNA sequence (Reik et al. 2001; Li 2002). These molecular modifications can lead to long-term variations in gene expression through stable gene silencing/activation. Increasingly, it is recognized that epigenetic marks provide a mechanistic link between environmental experiences, such as stress, social interactions, nutrition, and toxicological exposures, and variation in a broad range of phenotypic outcomes, including disease risk (Jirtle and Skinner 2007; Champagne 2010; Verhoeven et al. 2010). Although the DNA sequence is stable and highly conserved within and across generations, epigenetic modifications have the potential to be dynamic throughout life and can be heavily influenced by external factors (Reik et al. 2001). Thus, external effects on the epigenome may alter patterns of gene expression, potentially giving rise to phenotypic diversity. Epigenetic modifications include chromatin remodeling, post-translational histone tail modifications, DNA methylation, and more recently have expanded to include noncoding RNA and microRNA gene regulation (Jablonka and Lamb 2005; Matzke and Birchler 2005; Milosavljevic 2011).

Although epigenetic effects have been proposed to be important for evolutionary processes (Jablonka and Lamb 2005; Feinberg and Irizarry 2010; Verhoeven et al. 2010; Danchin et al. 2011), empirical data supporting the transgenerational inheritance and fitness consequences of epigenetic marks are still quite limited. Studies of naturally living populations are crucial to the empirical testing of epigenetic theories (Bossdorf et al. 2008; Ledón-Rettig et al. in press). The acquisition and analysis of DNA from naturally living populations can be problematic, however, as noninvasive techniques must often be used. In recent years, the collection of fecal samples and extraction of genomic DNA has been validated across a wide variety of taxa, and has been critical in the advancement of molecular ecology (Beja-Pereira et al. 2009; Perry et al. 2010). In principal, these methods may also be used to obtain the molecular samples necessary for DNA methylation analyses.

The use of bio-available tissues for epigenetic analyses faces a unique problem because epigenetic marks can be cell specific and are thought to maintain tissue-specific patterns of gene expression among differentiated cell types. For example, tissue-specific differentially methylated regions (T-DMRs) occur both within (De Bustos et al. 2009) and outside of (Eckhardt et al. 2006) CpG islands. For molecular ecologists, this specificity poses one of the largest obstacles to using non-invasive samples for epigenetic analysis. That is, there is no a priori reason to expect that peripheral samples (i.e., from feces, saliva, or other available tissues) will contain biologically relevant DNA methylation data and there may be concordance between tissues for some loci (Waterland and Jirtle 2003), but not others. Thus, it is necessary to validate candidate genes from peripheral samples to ensure that they predict biologically relevant methylation data in key target tissues (e.g., brain, liver, spleen).

As the first known attempt to explicitly investigate the epigenetic relationship between target tissue and bio-available samples for use in evolutionary research, this study compared DNA methylation levels of the NGFI-A (nerve growth factor-inducible protein A) binding site of the glucocorticoid receptor gene (*Nr3c1*) across hippocampal and fecal samples in the mouse (*Mus musculus*). This locus was chosen because it is highly conserved across mammals (Turner and Muller 2005). Additionally, methylation at this locus has been previously shown to vary across individuals as a function of the experience of variation in postnatal (PN) maternal environment (Weaver et al. 2004). The goals of this study were to (1) determine the concordance in CpG methylation patterns of the *Nr3c1* gene in hippocampal and fecal samples, (2) identify CpG sites within this genomic location where DNA methylation differed across individuals as a function of PN experience, and (3) to assess whether this differential methylation was detectable in both hippocampal and fecal samples.

## Methods and Materials

### Animals and assessment of maternal behavior

All subjects were Balb/c laboratory mice (*M. musculus*) purchased from Jackson Laboratories, and all procedures were undertaken in accordance with guidelines of the NIH regarding the Guide for the Care and Use of Laboratory Animals and with the approval of the Institutional Animal Care and Use Committee (IACUC) at Columbia University. Animals were housed at Columbia University on a reverse 12-h-dark and 12-h-light cycle, under a constant temperature of 21°C and 55% humidity. All animals were provided ad libitum access to water and chow diet. Behavioral observations took place during the dark period of the light cycle, this being the period when mice are most active.

Following a 2-week habituation period, group-housed (2–3/cage) female mice were mated for 14–16 days. Females were singly housed at approximately gestational day 18 and then monitored daily to determine the timing of birth of the litter. On the day of birth at PN day 0, pups were weighed and counted and then dams and litters were left undisturbed throughout the maternal observation period. As litter size was not standardized, we used litter size/weight as a covariate in the statistical analyses.

The procedure for assessing variation in maternal behavior has been previously described (Champagne et al. 2007). Briefly, maternal behavior of all dams (*n* = 14) was scored from day 1 through day 6 postpartum, and the observers were trained to a high level of inter-rater reliability (i.e., >0.90). Four observations were conducted daily, with two within 4 h of the onset of the dark cycle, and two near the end of this period. Within each observation period, the behavior of each dam was scored every 3 min (20 observations/period × 4 periods per day = 80 observations/mother/day = 480 observations per dam over the 6 days). The following behaviors were scored: mother licking and grooming any pup, mother in nursing posture over pups, nest-building, self-grooming, eating, and drinking. The calculated percent frequency of nursing and pup licking/grooming was used in subsequent analyses. This percentage represents the total times the behavior was observed divided by the total number of observations conducted. Both overall maternal care (PN1-6) and maternal care occurring on PN1 were included as predictors of offspring outcomes. Due to the overall low levels of pup licking/grooming in this sample (*M* = 3.83%, SD = 1.93%), nursing frequency (*M* = 31.06%, SD = 2.03%), which ranged from 11.67% to 49.58% in this sample, was used as the primary measure of maternal care (although pup licking/grooming was used as a statistical covariate).

Pups were weaned at PN day 28 and housed in same-sex groups of 3–4/mice per cage. A total of 22 (*n* = 22) pups were included in the initial analysis (*n* = 12 female; *n* = 10 male). Pups were derived from a total of 14 litters with only four litters sampled more than once (2–4 pups).

### Tissue collection

At PN day 35, mice were singly housed for 12 h and subsequently sacrificed. Whole-brain and fecal boli samples were collected (Columbia University), snap frozen on dry ice, and stored in a −80°C freezer prior to shipment to the University of Michigan (UM). Upon receipt at UM, brain and fecal samples were stored at −80°C until the time of dissection or DNA extraction.

### Brain dissection

All brains underwent gross dissection for a hippocampi-enriched block of tissue. This was achieved using a mouse brain slicer matrix (Zivic Instruments, Pittsburgh, PA). Brains were brought to −20°C and stored in a microtome chamber until the moment of dissection. The brain slicer was chilled to −80°C on dry ice and was removed from dry ice for each dissection. Each brain was placed in the matrix, in a dorsal–ventral orientation. Four-millimeter slices were created using razor blades inserted into the matrix. Slices were removed and placed back onto dry ice. Slices containing the dorsal hippocampus were visually identified by comparisons to a mouse brain atlas (Paxinos and Franklin 2004). This slice (coronal plane) was further dissected with just a razor blade to remove nonhippocampal tissues dorsally, ventrally, and laterally. This hippocampal-enriched area was then bisected along the midline and tissue halves placed in separate aliquots. Additional aliquots of forebrain, hindbrain, and extra-hippocampal regions from the original slice were all placed in 1.5-mL conical microcentrifuge tubes and returned to −80°C for storage.

### DNA extraction

One aliquot of hippocampal tissue was removed from −80°C and thawed to room temperature. DNA was extracted using the Qiagen DNeasy kit (Valencia, CA), according to the manufacturer's manual spin-column protocol. Samples were eluted into 200 μL of TE buffer. Two fecal pellets were thawed to room temperature, and DNA was extracted using the Maxwell-16 Instrument (Promega Corporation, Madison, WI). Fecal DNA samples were eluted into 350 μL of TE buffer.

### Pyrosequencing of bisulfite-treated tissue samples

**Figure 1:**
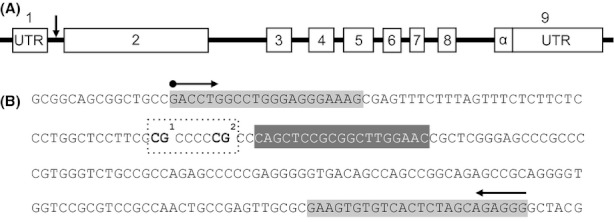
(A) Genomic organization (not to scale) of the mouse *Nr3c1* gene, showing the location of the NGFI-A binding site (arrow) targeted by pyrosequencing primers (B). Architecture of *Nr3c1* NGFI-A binding site. The location of the forward and reverse primers is indicated by arrows and gray highlighting, and the sequencing primer is identified by dark gray. The forward primer was biotinylated, as shown by the dot at the end of the arrow. The NGFI-A binding site is indicated in the box (dashed line). Sites referred to as CpG 1 and CpG 2 are indicated.

Bisulfite pyrosequencing (Tost and Gut 2007) was used to obtain methylation values for two CpG sites within the NGFI-A binding region of *Nr3c1* (Fig. 1). Samples were bisulfite converted using the Qiagen Epitect kit. Conversions were executed according to the manufacturer's automated protocol for the Qiagen Qiacube. Samples were then amplified using the following primers: (5′ FBIO-GAT TTG GTT TGG GAG GGA AAG 3′) and (5′ R-CCT CTA CTA AAA TAA CAC ACT TC 3′), and Qiagen *HotStarTaq* master mix with an annealing temperature of 55°C. Polymerase chain reaction (PCR) products were resolved by gel electrophoresis prior to pyrosequencing. This step revealed three fecal samples that failed to amplify, resulting in a reduced sample size for fecal methylation analysis (hippocampus, *n* = 22; fecal, *n* = 19). To ensure that tissue-specific differences in CpG methylation were not the consequence of this disparity in sample size, all analyses comparing effects in hippocampal versus fecal samples were restricted to individuals in which both tissues were available (*n* = 19). PyroGold reagents (Qiagen) were used to prepare samples for pyrosequencing according to the manufacturer's instructions. CpG methylation was quantified using the Pyromark MD system and Pyro Q-CpGt 1.0.9 software (Qiagen). All samples (hippocampal and fecal) were processed on three separate runs, however, only the third contained duplicates. Only samples from the third run were used to test duplicate reliability. The Pyromark MD software has a number of built-in quality controls, meaning that every sample either “passes,” “fails,” or “conditionally passes.” Methylation values were only taken from samples that “passed” at both CpG sites.

### Statistics

All data analysis was conducted with SPSS (version 19.0 for PC), and the statistical threshold for all tests was set to *P* <0.05. Duplicate reliability (a quality control measure) was assessed using a paired *T*-test to compare methylation values of only the samples where both duplicates “passed” (*n* = 20 sets of *n* = 41 total sets). DNA methylation percentages were subsequently averaged within individuals across duplicates and runs for each CpG site and tissue. One-way repeated measures analysis of variance (ANOVA) was used to determine the potential impact of sex and tissue type on average *Nr3c1* DNA methylation. *T*-tests were used to determine differences in methylation across individual CpG sites within each tissue (i.e., average% methylation at site 1 versus 2 in hippocampal/fecal tissue). Pearson correlation coefficients were used to determine the association between CpG methylation at CpG sites 1 and 2 and between percent DNA methylation in brain and fecal samples (i.e., degree of correlation in % methylation at sites 1 and 2 in hippocampal/fecal tissue; correlation between brain and fecal CpG methylation levels at site 1, site 2, and the average of sites 1 and 2). A multiple weighted least squares regression model was used to examine the relationship between maternal and life history variables (nursing, pup licking, litter size, and litter weight) and DNA methylation levels at each of the two CpG sites measured across both tissues (hippocampus and fecal boli). The association between methylation levels in fecal boli and those in the hippocampus was analyzed using multiple weighted least squares regression models that were run in a stepwise fashion. First, methylation in the boli alone was used to predict hippocampal methylation followed by a second model that included maternal care variables (nursing, licking). The change in variance explained in the subsequent model (*R*^2^-change) was tested using an *F*-test.

## Results

### Duplicates

**Figure 2:**
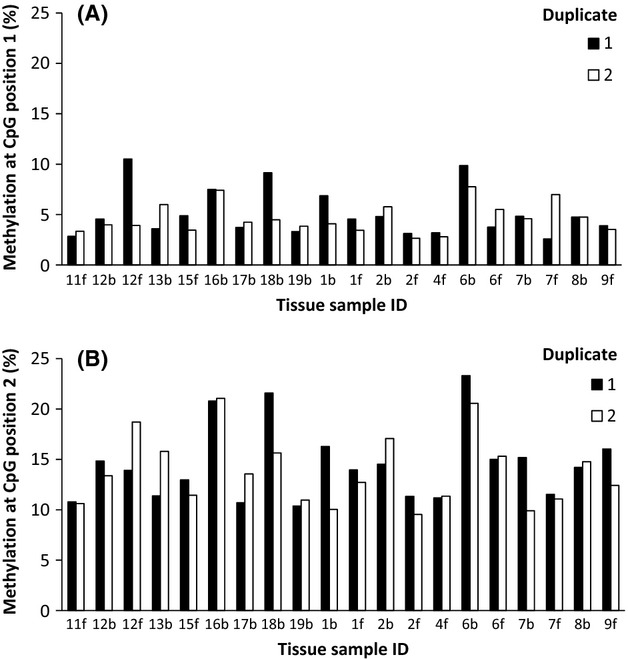
Comparison of pyrosequencing run #3 duplicates for (A) CpG position 1 and (B) CpG position 2.

Comparison of CpG methylation levels obtained in the duplicates of the assay was conducted to determine the stability of the assay and the consistency of the percent methylation levels. At CpG site 1, there was no significant difference in methylation between the first duplicate (*M* = 5.11%, SD = 2.38) and the second duplicate (*M* = 4.62%, SD = 1.68; *t*(20) = 0.92, *P* = 0.37; Fig. 2A). Similarly, there was no significant difference at CpG site 2 between the first duplicate (*M* = 14.40%, SD = 3.71) and the second duplicate (*M* = 13.79%, SD = 3.49; *t*(20) = 1.01, *P* = 0.32; Fig. 2B). As duplicates did not show any difference, the methylation values across duplicates and across all three runs were averaged to obtain site and tissue-specific methylation values for each sample.

### Overall CpG methylation

Average percent CpG methylation was not found to vary as a function of sex (*F*(1, 18) = 0.06, n.s.) or tissue type (*F*(1, 18) = 0.43, n.s.) (Table 1). In both brain (*t*(18) = 21.07, *P* < 0.001) and fecal boli samples (*t*(18) = 8.79, *P* < 0.001), average % DNA methylation was found to be elevated at CpG site 2 compared with CpG site 1. In brain tissue, there was a highly significant correlation between CpG methylation levels at site 1 and 2 (*r*(19) = 0.93, *P* < 0.001). However, in fecal boli, this concordance in CpG methylation at sites 1 and 2 was not significant (*r*(19) = 0.29, n.s.).

**Table 1:** Percent DNA methylation within the *Nr3c1* promoter (mean ± SEM)

Tissue	Sex	CpG site 1	CpG site 2
Brain	Male	5.07 (0.27)	13.80 (0.73)
	Female	5.16 (0.37)	13.41 (0.94)
Fecal boli	Male	5.33 (0.46)	14.58 (0.65)
	Female	6.25 (1.58)	13.21 (0.61)

### Maternal care and CpG methylation

**Figure 3:**
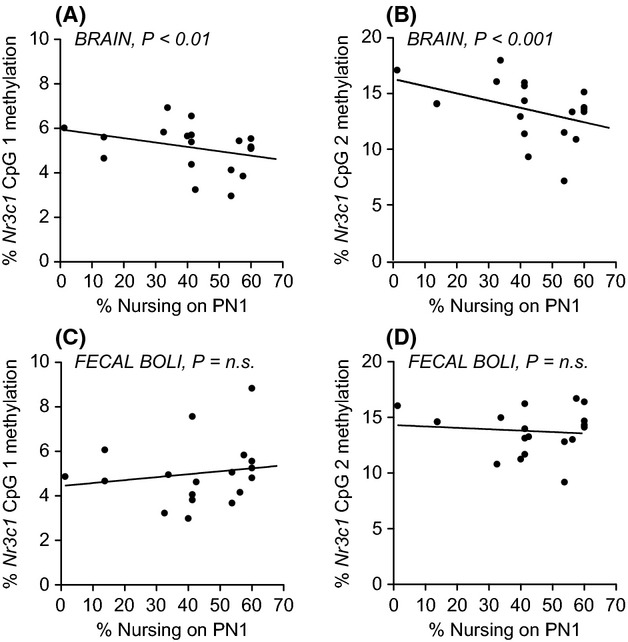
Correlation between postnatal nursing on PN1 and CpG methylation in brain and fecal boli samples. Note that indicated *P*-values reflect the significance of the regression model adjusting for the effects of pup licking, litter size, and litter weight.

Frequency of nursing and pup licking at PN1 and across the first week postpartum were found to be significantly correlated (PN1: *r*(22) = 0.50, *P* < 0.05; PN1-6: *r*(22) = 0.77, *P* < 0.001). Although overall nursing (PN1–PN6) was not a significant predictor of *Nr3c1* methylation levels, nursing frequency on PN day (PN1) was negatively associated with juvenile hippocampal *Nr3c1* methylation levels of CpG site 1 (*t*(18) = 3.61, *P* < 0.01; Fig. 3A) and at CpG site 2 (*t*(18) = 4.75, *P* < 0.001; Fig. 3B) after accounting for pup licking, litter size, and litter weight. This effect of maternal nursing on *Nr3c1* methylation appears to be tissue specific, as there was no significant relationship between PN maternal nursing and levels of *Nr3c1* methylation in the fecal boli at either of the CpG sites measured (fecal CpG site 1: *t*(18) = 0.09, n.s.; fecal CpG site 2: *t*(18) = 0.80, n.s.; Fig. 3C and D).

### Predicting hippocampal methylation levels using fecal boli methylation

**Figure 4:**
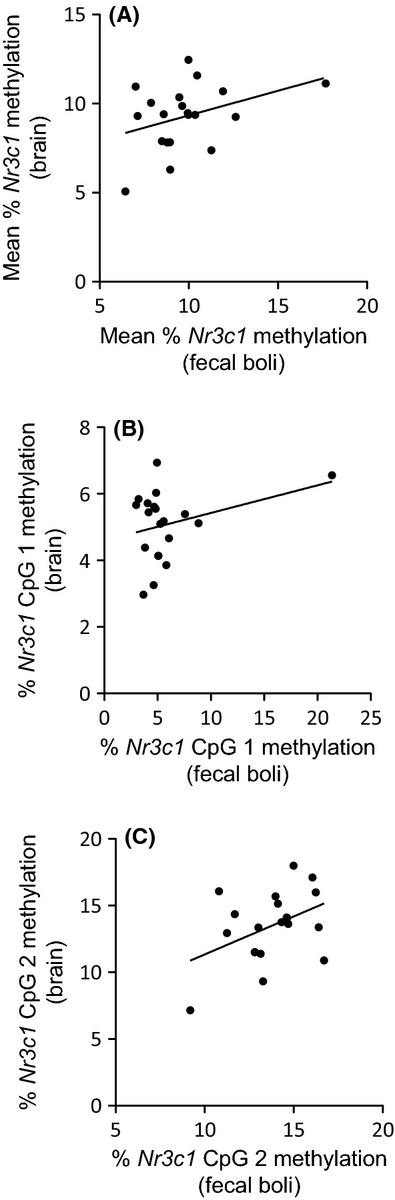
Correlation between *Nr3c1* CpG methylation in fecal boli and hippocampal samples. (A) Average% methylation across sites 1 and 2 (*r* = 0.36, n.s.), (B) site 1 (*r* = 0.31, n.s.), and (C) site 2 (*r* = 0.42, *P* = 0.08) correlations.

Average *Nr3c1* methylation levels in fecal boli was found to be a poor predictor of *Nr3c1* methylation levels in the hippocampus (*t*(18) = 1.60, n.s.) and the within-individual correlation in percent methylation between these tissues was found to be nonsignificant (see Fig. 4). However, a significant relationship between the average methylation levels within brain and fecal boli (averaged across CpG sites 1 and 2) was found to emerge after controlling for the maternal nursing and licking received during the first PN day (*t*(18) = 2.21, *P* < 0.05). Thus, CpG methylation levels in boli predicted those in the brain only after accounting for PN maternal care, variables which contributed significantly to the overall model (*R*^2^-change = 0.43, *F*(2, 15) = 7.49, *P* < 0.01). In other words, methylation levels in fecal boli were only a good predictor of those in the brain after holding early-life experiential variables constant. This was also marginally true for the relationship between specific CpG sites in the boli and their corresponding CpG sites in the brain. Although methylation levels of the boli at individual sites failed to predict methylation at the corresponding site in the hippocampus (CpG site 1: *t*(18) = 1.35, *P* = n.s; CpG site 2: *t*(18) = 1.90, *P* = 0.08), subsequent analyses revealed that specific CpG sites in the boli became marginally good predictors of methylation at their corresponding CpG sites in the hippocampus (CpG site 1: *t*(18) = 1.84, *P* = 0.09; CpG site 2: *t*(18) = 1.86, *P* = 0.08) only after accounting for PN nursing and licking (CpG site 1: *R*^2^-change = 0.31, *F*(2, 15) = 3.84, *P* < 0.05; CpG site 2: *R*^2^-change = 0.40, *F*(2, 15) = 7.03, *P* < 0.01).

## Discussion

This study is the first known attempt to explicitly validate the use of fecal samples for ecological methylation analyses. The results indicate that an element of the social environment, maternal behavior exhibits an effect on brain DNA methylation. These findings are supported by previous work showing maternally mediated methylation differences in the NGFI-A binding region of *Nr3c1* in the rat (Weaver et al. 2004). Novel to this study, however, we have shown that this socially mediated methylation pattern is specific to the brain, and is not detected in fecal DNA. Moreover, *Nr3c1* CpG methylation within individuals was not found to be correlated between brain and fecal DNA unless the effects of PN maternal care were accounted. As such, the tissue-specific effects of maternal care may account for the lack of concordance between DNA methylation levels between central (hippocampal) and peripheral (fecal) tissue. As maternal care increases *Nr3c1* methylation in the hippocampus, but not in fecal samples, this tissue-specific effect appears to induce increased within-individual divergence in *Nr3c1* methylation. Although the *Nr3c1* gene is highly conserved across vertebrates (Thornton 2001), it is not present in bacteria. Therefore, it is unlikely that the locus-specific primers spuriously amplified non-target DNA. Additionally, the fecal samples amplified successfully with the primers specific to the murine NGFI-A locus, indicating sufficiently high DNA quality, even after bisulfite conversion. Thus, the CpG methylation characteristics in fecal samples cannot be attributed to low DNA quality.

These results highlight the importance of validating functional epigenetic results in bioavailable tissues. Although epigenetic studies analyzing DNA methylation in peripheral tissues from wild populations have only just begun to emerge (Schrey et al. 2012), numerous epidemiological studies have been conducted using blood as a surrogate biomarker. Blood is a relatively noninvasive sample tissue that yields high-quality DNA, but is not necessarily considered a primary or central tissue. As such, results that indicate methylation effects in blood (Oberlander et al. 2008; Kinnally et al. 2011) must be interpreted cautiously if their functional effects are postulated to take place in the brain. Therefore, with our results in mind, it will be important to conduct proof-of-principle studies validating functional epigenetic results with noninvasive samples. Moreover, our results indicate that environmental exposures may increase the discordance in DNA methylation levels between different tissues – a phenomenon that merits further investigation.

Despite this finding of a lack of concordance between central and peripheral DNA methylation patterns at one epigenetically labile locus, it is still extremely important for molecular ecologists to obtain DNA methylation information from naturally living populations. Rather, we encourage an increased research effort toward the comparison of methylation patterns across tissues. Specifically, comparisons with multiple relevant peripheral tissues such as saliva and feces as well as increased sample sizes to assess tissue concordance will be fruitful for molecular ecologists, behavioral biologists, and psychologists. In addition to these kinds of validation studies, alternative methods and nonfunctional loci remain an alternative option to studying epigenetic patterns in wild populations. In mice (Weinhouse et al. 2011) and humans (Waterland et al. 2010), a burgeoning literature is identifying metastable epialleles (MEs). These loci represent regions where stable methylation patterns are established in early embryonic development, resulting in concordant methylation patterns across all germ layers. Despite being similar across tissues, MEs vary across individuals. As such, they act as biosensors for individual differences in DNA methylation, and are detected across tissue from the three germ layers (Waterland and Jirtle 2003; Dolinoy et al. 2006). In some ways, MEs are ideal for studying natural populations, but there are drawbacks. MEs require extensive validation (Weinhouse et al. 2011), and may not regulate a genomic region of functional interest.

Another option available to molecular ecologists is the LUminometric Methylation Assay (LUMA; Karimi et al. 2006). This methylation-dependent restriction enzyme-based assay provides a measure of methylation at CCGG sites throughout the entire genome regardless of location, representing the degree to which the genome is globally methylated. This assay incorporates combined DNA cleavage by methylation sensitive restriction enzymes, similar to the methylation-sensitive amplified fragment-length polymorphism (MS-AFLP) approach, but quantifies the resulting methylation values by polymerase extension assay on a MD 96 Pyrosequencing™ platform. In this way, experiments can be scaled up and automated. Additional benefits of the LUMA assay include: (1) minimal starting DNA as bisulfite conversion is not necessary, (2) time and cost savings compared with other methylation assays, and (3) the ability to measure methylation in species without a reference sequence, where sequencing-based methylation assays cannot (Head et al. 2012). This approach can be useful for detecting large-scale stimuli (such as environmental toxicants), and may therefore have high utility for conservation efforts. However, this method yields no functional information; it is not possible to test hypotheses about specific genes or biological pathways. Additionally, the LUMA-based approach must be applied to tissues that contain only target-species DNA. Fecal samples cannot be used because they contain DNA from bacteria and plants, often in much higher quantity than the target-species DNA.

## Conclusion

This study presents a novel tissue-specific comparison of methylation between brain and feces, specifically designed for molecular ecologists working with noninvasive samples. We found that fecal DNA methylation profiles at the NGFI-A binding site of *Nr3c1* were not correlated with DNA methylation profiles in hippocampal tissue and were not significantly influenced by postpartum maternal behavior, suggesting that molecular ecologists should exercise caution when applying epigenetic methods to noninvasive samples. Tissue specificity is a pervasive signature of the epigenome, and must be accounted for when analyzing peripheral tissues. Conversely, these results are specific to only two CpG sites in the mouse genome within two tissue types, and may not be generalizable to other loci, other tissues, or other species. In addition, epigenetic effects may vary dependent on the type of environmental exposure, and thus the effects we observe in response to maternal care may not generalize to other types of experiences/exposures. Further studies will be needed to identify the functional regions that can be detected in noninvasive samples. Where explicit comparisons of central and peripheral tissues are not possible, other methods can be utilized. Assaying MEs, or use of global methylation approaches such as the LUMA assay, may be fruitful avenues for further molecular ecology research.
